# An Acoustic Tracking Approach for Medical Ultrasound Image Simulator

**DOI:** 10.1007/s40846-017-0258-9

**Published:** 2017-06-21

**Authors:** Po-Heng Chen, Kai-Sheng Hsieh, Chih-Chung Huang

**Affiliations:** 1 Department of Biomedical Engineering, National Cheng Kung University, No 1, University Road, Tainan City, 70101 Taiwan; 2 Division of Critical Care Medicine, Department of Pediatrics, Kaohsiung Chang Gung Memorial Hospital and Chang Gung University College of Medicine, Kaohsiung, 833 Taiwan

**Keywords:** Ultrasound simulation system, Ultrasound examination, Acoustic tracking, Air-coupled ultrasound transducer

## Abstract

Ultrasound examinations are a standard procedure in the clinical diagnosis of many diseases. However, the efficacy of an ultrasound examination is highly dependent on the skill and experience of the operator, which has prompted proposals for ultrasound simulation systems to facilitate training and education in hospitals and medical schools. The key technology of the medical ultrasound simulation system is the probe tracking method that is used to determine the position and inclination angle of the sham probe, since this information is used to display the ultrasound images in real time. This study investigated a novel acoustic tracking approach for an ultrasound simulation system that exhibits high sensitivity and is cost-effective. Five air-coupled ultrasound elements are arranged as a 1D array in front of a sham probe for transmitting the acoustic signals, and a 5 × 5 2D array of receiving elements is used to receive the acoustic signals from the moving transmitting elements. Since the patterns of the received signals can differ for different positions and angles of the moving probe, the probe can be tracked precisely by the acoustic tracking approach. After the probe position has been determined by the system, the corresponding ultrasound image is immediately displayed on the screen. The system performance was verified by scanning three different subjects as image databases: a simple commercial phantom, a complicated self-made phantom, and a porcine heart. The experimental results indicated that the tracking and angle accuracies of the presented acoustic tracking approach were 0.7 mm and 0.5°, respectively. The performance of the acoustic tracking approach is compared with those of other tracking technologies.

**Electronic supplementary material:**

The online version of this article (doi:10.1007/s40846-017-0258-9) contains supplementary material, which is available to authorized users.

## **I**ntroduction

Medical ultrasound examinations have become essential for diagnostic, therapeutic, and surgical procedures in hospitals and other clinical environments. An ultrasound system can provide high-resolution cross-section images of the abdomen and its internal organs that are useful in many clinical applications, such as cardiology, obstetrics, and gynecology, as well as of the breast, thyroid, and vascular and musculoskeletal systems. However, the efficacy of an ultrasound examination is highly dependent on the skill of the operator. Misjudgment can occur if the operator lacks sufficient experience, particularly when detecting rare disorders. It is therefore very important that junior sonographers receive a training course in ultrasound examinations [1]. According to the recommendations from the American College of Cardiology and the American Heart Association, trainees who intend performing transthoracic echocardiography in adult patients need 12 months of training to achieve proficiency, which includes a minimum of 300 examinations and the interpretation of 450 Doppler examinations [2, 3]. The requirement for such a large number of examinations represents a large burden on both hospitals and trainees, and moreover it may still be insufficient for ensuring that trainees are able to detect cases of rare disorders. This situation has prompted proposals for the development of an ultrasound simulation system for assisting ultrasound examination training and education.

An ultrasound simulation system can provide very effective training without impacting patient safety because it can simulate highly accurate scenarios, uses very realistic tools, and affords opportunities to manage patient complications [4]. The high dependence of the outcome of an ultrasound examination on the probe-handling skill of the operator means that the system for tracking the probe position is a very important component of an ultrasound simulation system [5]. Several tracking approaches have been developed in the last decade, including optical tracking (OT), electromagnetic tracking (EMT), and inertial tracking (IT). Any tracking system can be characterized by its sampling rate, sensitivity, precision, working range, and degrees of freedom (DoF), and each tracking approach has its own advantages and disadvantages.

The OT approach for ultrasound simulation systems provides high accuracy, high update rates, and a relatively large workspace [6–8]. For instance, an ultrasound education ultrasound simulation system for the abdominal region that integrates Web cameras and several planar optical markers printed on paper sheets has been reported [9]. However, OT requires the line of sight to be maintained between the tracking device and the probe, and so the presence of any obstacle, ambient light, or infrared radiation between the detector and markers degrades the performance when using the OT approach. In EMT, a magnetic field sensor placed on a probe measures electrical currents induced as the probe moves in a magnetic field generated by an electrical field generator [10–17]. However, EMT has the disadvantage that signals from sources such as power cables and surrounding instruments can interfere with the tracking system signals and thereby impair the tracking accuracy. This makes it challenging to use EMT in an environment where various metallic objects are moving around in the magnetic field generated by the electrical field generator. However, EMT is still the most popular approach for tracking the probe position in ultrasound simulation systems [18–22]. The IT approach is a navigation technique that employs accelerometers and gyroscopes to track the position and orientation of an object relative to a known starting point. While IT is cheaper than other approaches, the large measurement error that accumulates over time is a major disadvantage for an ultrasound simulation system [23]. The acoustic method has been also used to track the probe position in a 3D space [24–32]. In this method, sound-emitting devices were mounted on the ultrasound probe, and the fixed microphones were mounted above the patient. The microphone was used to receive the sound signals from the emitting devices as the probe is moving. The position and orientation of the probe then was determined by measuring the speed of sound in air between each emitter and microphone. However, the microphone must be placed over the patient and must close enough to the emitter in order to obtain a good signal-to-noise ratio. In addition, many studies used several tracking approaches for 3D ultrasound imaging [33–36].

The present study investigated a novel acoustic tracking approach for use in a medical ultrasound simulation system. Air-coupled ultrasound elements are embedded in the front of a sham ultrasound probe for transmitting the acoustic signals, and the position and orientation of the sham probe are tracked by receiving the acoustic signals using 2D air-coupled ultrasound elements. After the position of the sham probe is identified by the acoustic tracking approach, its corresponding ultrasound image is displayed according to the position of a real ultrasound examination in the image database as obtained previously via the mechanical scanning of subjects. The validity of this approach was verified in phantom and in vitro porcine heart experiments, and the system performance was compared with those of several commercial ultrasound simulation systems.

## Materials and Methods

### System Description

**Fig. 1:**
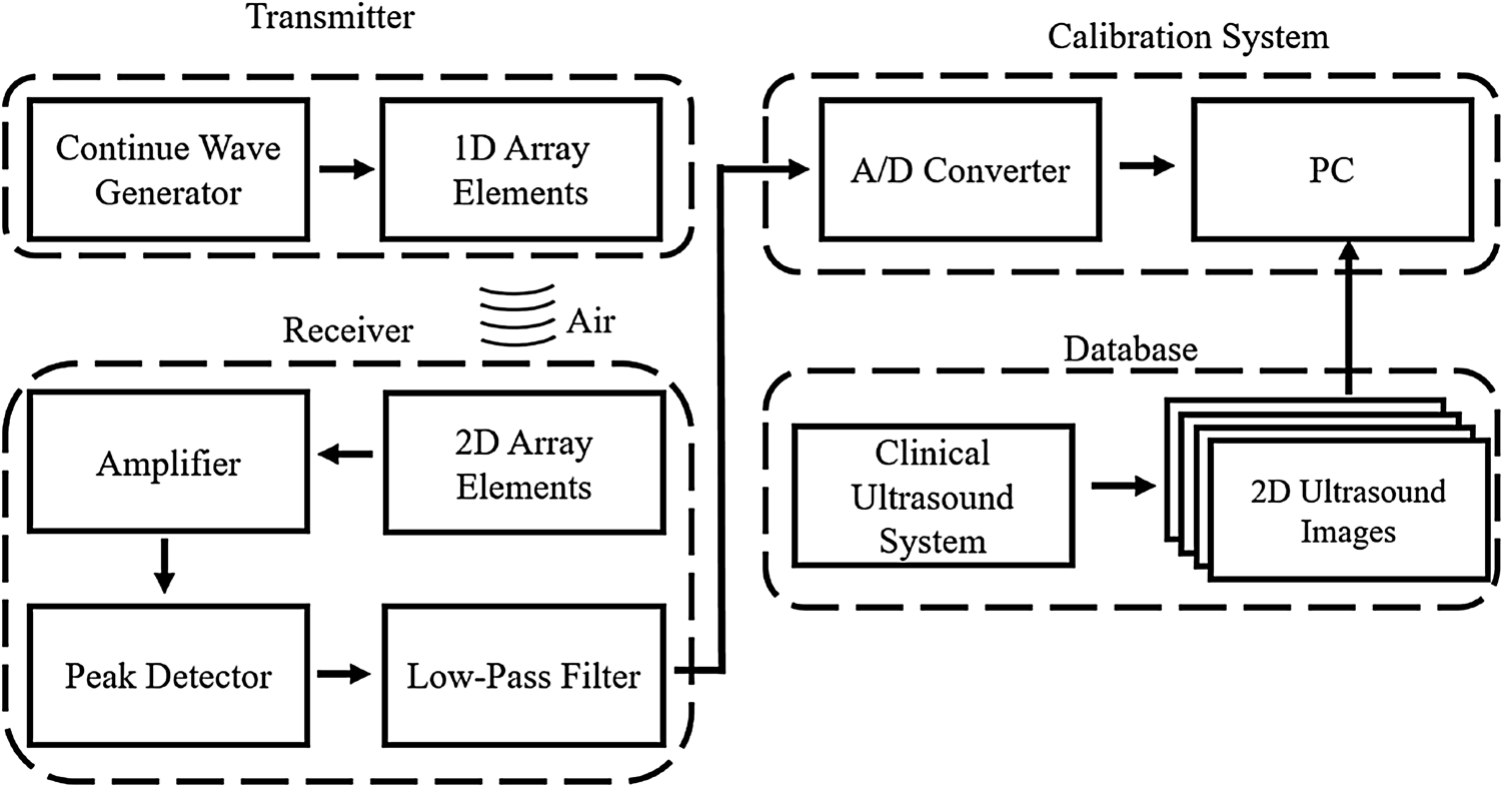
Block diagram of the ultrasound simulation system

**Fig. 2:**
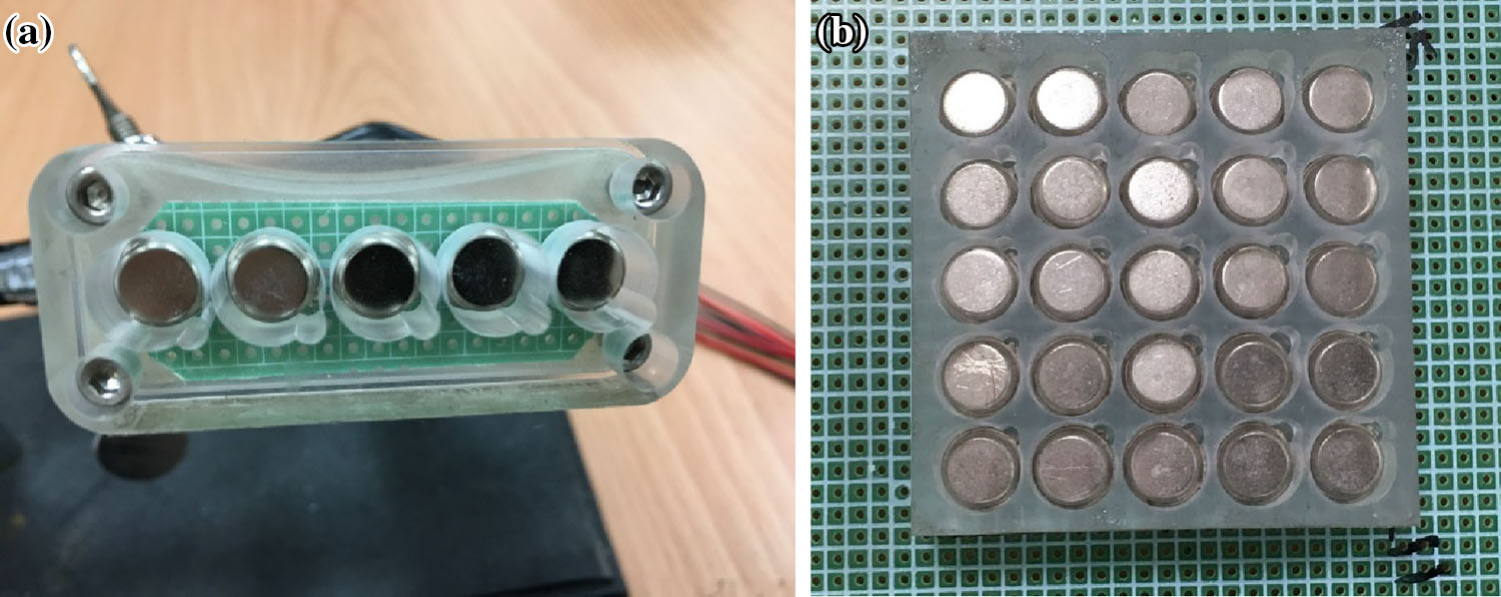
Photographs of the 1D air-coupled transmitting elements (**a**) and the 5 × 5 2D array of receiving elements (**b**)

Figure 1 shows a block diagram of the ultrasound simulation system based on the acoustic tracking approach. Air-coupled ultrasound elements were used for transmitting and receiving the acoustic signals (respectively 400ET080 and 400ER080, Prowave, Taipei, Taiwan). The ultrasound frequency of the air-coupled element is 40 kHz. The bandwidth of the transmitting element is 1.5 kHz. Five transmitting elements were embedded as a 1D array in front of a sham ultrasound probe, and a five-channel continuous-wave generator with an output voltage of 15 Vpp was designed for exciting the elements. The sham ultrasound probe was made using a 3D printer according to the shape of a commercial ultrasound probe (12L5, Terason, Massachusetts, USA). Moreover, each element was isolated by pieces in order to reduce the interference from adjacent elements, as shown in Fig. 2(a). A 5 × 5 2D array of receiving elements was used to receive the acoustic signals from the moving transmitting elements (in the sham ultrasound probe), as shown in Fig. 2(b). Each received signal passed through an independent amplifier, peak detector, and low-pass filter. The peak detector converted the input sine-wave signal into a DC voltage and thereby decreased the frequency of signal changes for the subsequent processing by an analog-to-digital converter. A first-order low-pass filter was used to remove the high-frequency noise. The 25 receiver elements were integrated on a single circuit board. A data acquisition module (USB 6343, National Instruments, Texas, USA) with 32 analog input ports was used to record the 2D DC voltage map in a personal computer (PC) via a USB interface; the sampling rate and resolution of the USB 6343 module are 500 kS/sec and 16 bits, respectively. A graphical user interface was designed in LabVIEW software (version 2013, National Instruments) to allow real-time observations of variations in the 25 received acoustic signals. The user interface also displayed 2D ultrasound images corresponding to the position of the sham probe. The delay time between the motion of the sham probe and the image displayed in the software is 0.05 s. The distance between receiving elements and the transmitting elements is 10 mm in this system. Polyurethane film covers the receiver unit, and the sham probe can be moved freely over it by the operator. Since the probe was contacted with the film during the scanning, the distance between receiving elements and the transmitting elements is a stable in this system.

### Position Tracking Procedure

**Fig. 3:**
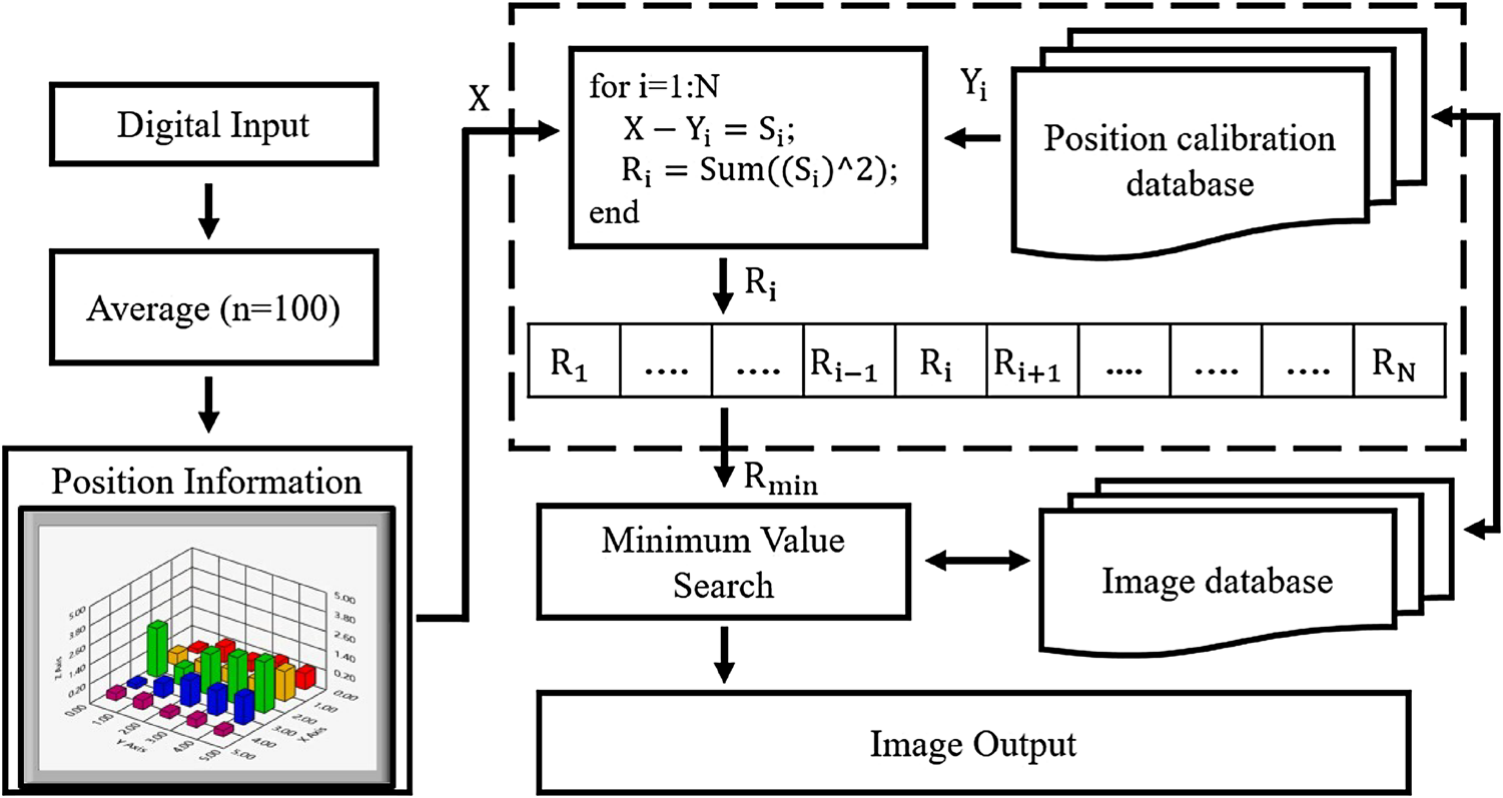
The procedure of the acoustic tracking approach

The sham probe was fixed on a four-axis motor platform and placed above the 2D array of receiving elements. As the sham probe is moved in the four-axis directions (X, Y, and Z directions, and the angle of inclination), the received acoustic signals and position information are recorded simultaneously in the position calibration database. Therefore, the spatial relationship between the estimated positions and orientations and their corresponding images was obtained as the image database. Figure 3 shows the procedure of the acoustic tracking approach. The digital input is the 5 × 5 DC voltage map that was transferred from the received acoustic signals. Since the digital input was obtained at a sampling rate of 500 kS/s, 100 frames of 2D array data were averaged as the position information. The height of a color bar (voltage map) in Fig. 3 represents the amplitude of the received acoustic signal at the defined position (X). The position information was used to match the position calibration database (Y_i_) for orientating the position of the sham probe. In the figure, N means the total position situation that was obtained from the motor platform. Each X was subtracted from Y_i_, with the results squared and summed across all values to find R_i_. The minimum value of R_i_ corresponds to the correct position in the calibration database, which means that the position of sham probe could be tracked effectively. The minimum value of Ri is close to 0. After R_i_ was calculated, minimum value search was used to find the value that closes to 0 between R_1_ and R_N_. As R_min_ was found, the system would find the image that was assigned in this position. For example, if the minimum value happened in R_50_, the system would capture image assigned in position 50 and display it on the monitor.

After the position and orientation of the sham probe are identified using the acoustic tracking approach, the real ultrasound image from a specific cross section of an organ is displayed on the system screen. The image database was obtained using a clinical ultrasound scanner (t3000, Terason) with a linear array probe (12L5). The probe was fixed on a four-axis motor platform so that it can be moved freely in 3D space by a motor controller. Open-source software is available for the Terason t3000 scanner to allow applications to be developed that run on the Windows operating system, which allows real-time ultrasound images to be acquired on a frame-by-frame basis in the PC as the probe is moved across the area of interest. The position information from the motor and image information from the scanner were integrated together in the image database. When the minimum value of R_i_ is determined, the corresponding ultrasound image is displayed on the user interface.

### Sample Preparation

**Fig. 4:**
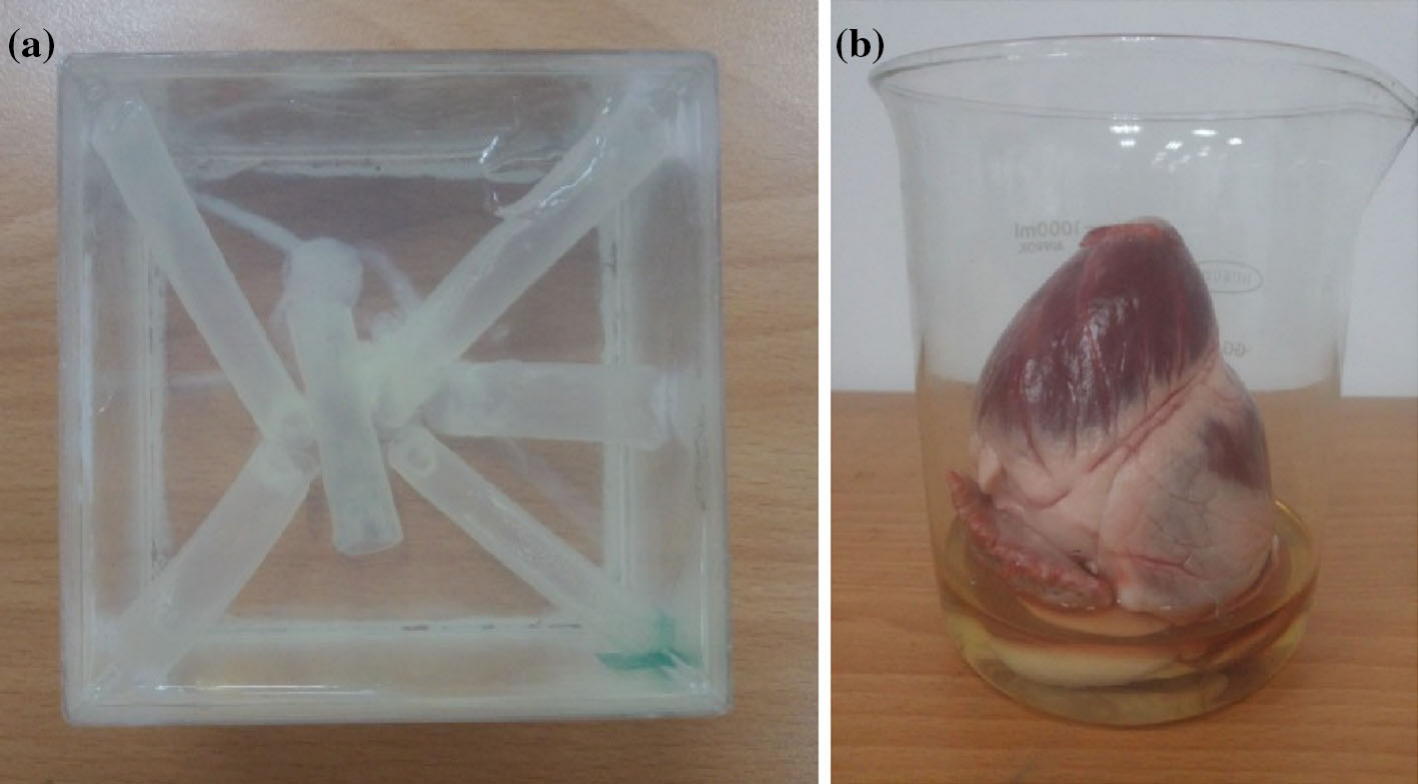
Photographs of the self-made phantom (**a**) and the porcine heart (**b**)

Three different subjects were used to verify the performance of the acoustic tracking approach for an ultrasound simulation system. The first subject was a commercial ultrasound phantom (040GSE, CIRS, Virginia, USA) which allowed for evaluations over ultrasound frequencies ranging from 2 to 15 MHz. Since the structure of this commercial ultrasound phantom is quite simple, a second phantom was constructed that had a complicated structure. Figure 4(a) shows a photograph of the self-made phantom, which contained several PVC tubes surrounded by 5% gelatin (type A, Sigma-Aldrich, Missouri, USA). The third subject was a porcine heart obtained from a local slaughterhouse, which was used for an in vitro experiment, as shown in Fig. 4(b). The gelatin solution was injected into the heart chambers in order to remove air from inside the atria and ventricles. After the gelatin solution had solidified, sticking and stitching were used to maintain the position of the porcine heart during the ultrasound examination.

## Results and Discussion

**Fig. 5:**
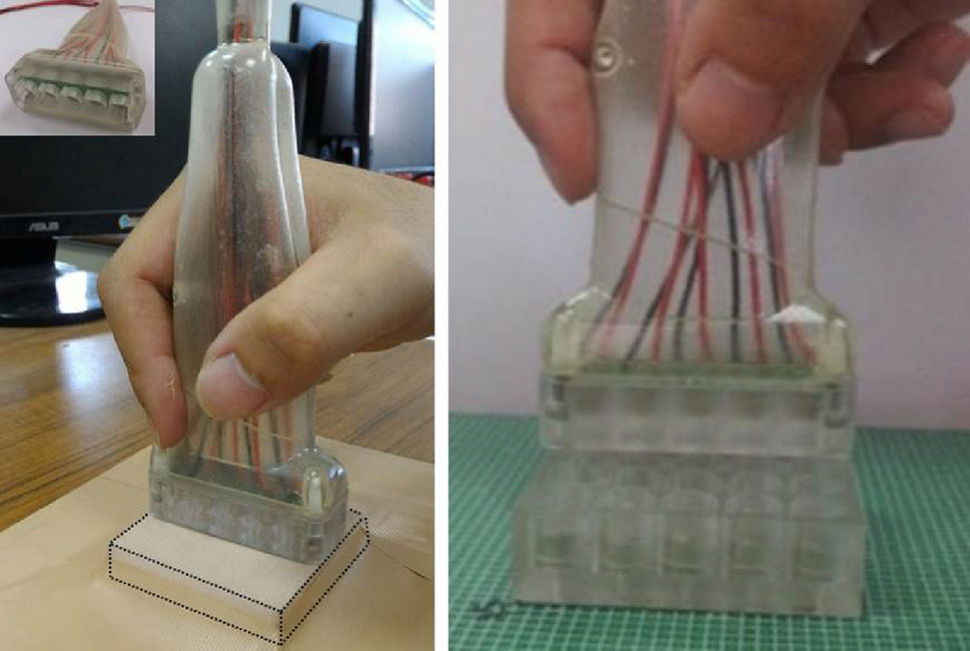
Photographs of the operation of the ultrasound simulation system, showing the receiver unit covered by polyurethane film

The size of the receiver unit is 55 × 55 mm, including the 25 elements. The receiving elements were fixed into an acrylic holder and soldered on a circuit board. The transmitting elements were connected to the continuous-wave generator. Figure 5 shows photographs of the ultrasound probe and how it is used. Polyurethane film covers the receiver unit, and the sham probe can be moved freely over it by the operator. Since the characteristic of each transmitting element is slightly different due to the fabrication procedure, the amplitude of the exciting voltage for each element needs to be adjusted individually to ensure that all five elements have the same output energy. Similarly, the gains of the receiving amplifiers were adjusted individually for each receiving element. The high sensitivity of the receiving elements means that even trembling of the hand causes variations of the received DC voltage map. Therefore, 100-times averaging is applied to the 2D voltage map before the position is identified. While increasing the number of averages makes the digital input more stable, this can also cause a delay due to the time required for the calculations, and so 100-times averaging was the best trade-off for our system. There are gaps between the elements in the receiver unit. However, these do not influence the acoustic signals received by the sham probe because the elements are not focused, and so the effect of acoustic wave dispersion means that the adjacent elements still can receive the signal from transmitting elements. The size of the receiving 2D array is 5.5 × 5.5 cm in current system. The predefined size of receiving unit depends on the applications. For example, the scanning area should be larger for abdominal ultrasound examination but smaller for carotid imaging. In present study, a 5 × 5 2D array of receiving elements were used for this feasibility study. However, the scanning area can be increased by adding more receiving elements.

**Fig. 6:**
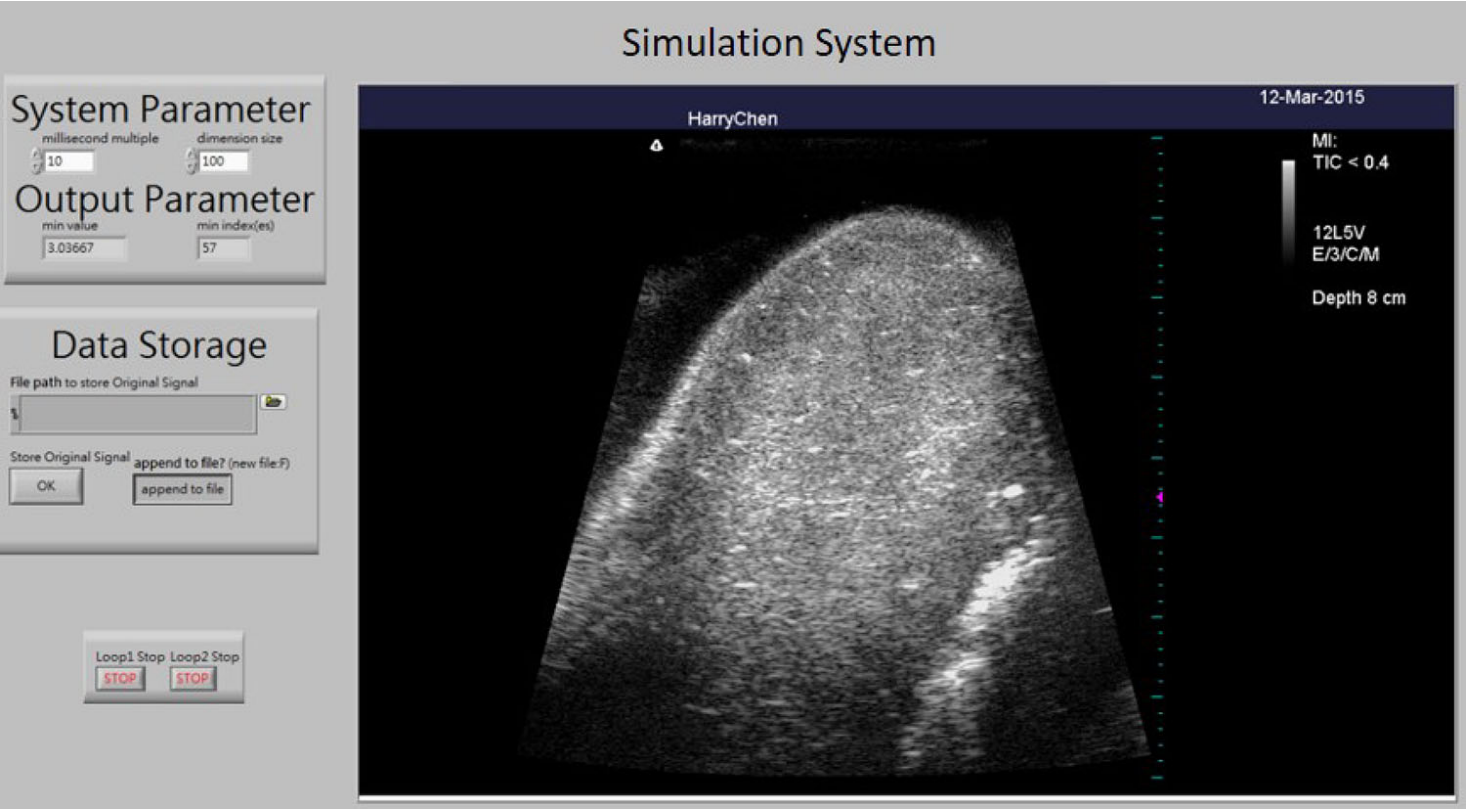
User interface of the ultrasound simulation system

**Fig. 7:**
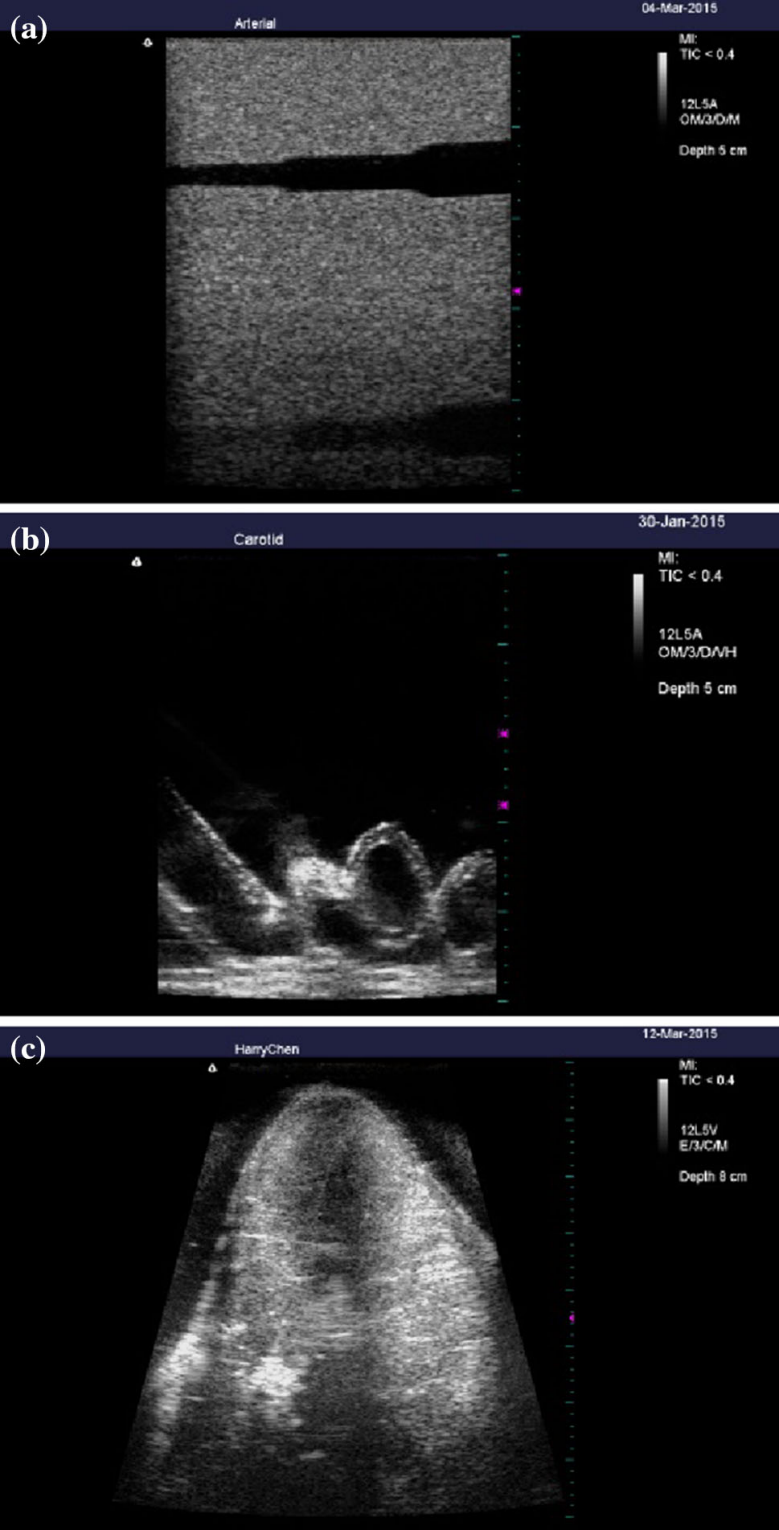
Operational results of the ultrasound simulation system for three different subjects: commercial ultrasound phantom (video 1) (**a**), self-made phantom (video 2) (**b**) and porcine heart (video 3) (**c**)

Figure 6 shows the user interface of the simulation system. As the sham probe is scanned over the receiver unit, the real time image of the object is displayed on the screen. Figure 7a–c show video screenshots from the simulation system for the commercial ultrasound phantom, self-made phantom, and porcine heart, respectively. In the videos displayed on the screen of the system, dynamic images are presented on the left side as the sham probe is swept over the receiver unit, while the corresponding real images obtained from the clinical ultrasound system by scanning a real subject are displayed on the right side. In short, the operator uses the sham probe to perform an artificial ultrasound examination of the receiver unit, while real images obtained from the subjects are displayed in real-time on the simulation system. The images in the simulator are of high quality since the image database comes from a clinical ultrasound system. While slight shaking sometimes appears on the left side of the videos, which can be attributed to the acoustic tracking approach being too sensitive in some cases, the images displayed in simulation system are generally well matched with those of the clinical system, as shown in Fig. 7. Use of porcine heart as the test organ because of it has an obvious structure in anatomy which including myocardium and four chambers. In the proposed ultrasound simulation system, the images were displayed as the ultrasound probe position was determined by the acoustic tracking approach. Since the images were recorded previously according to different clinical situations, the frame rate is not determined by the tracking system. In other words, as the position and orientation of the probe was determined by proposed system, the corresponding pre-recorded images were displayed on the screen. The amount of database depends on the application as well. Take the porcine heart experiment for example, the amount of images collected are 78 images per lateral and axial motion, and 720 images per rotation motion. 3D ultrasound image can also be applied into this system. But we used 2D image first for testing system performance in this study.

While the experimental results showed that the simulation system performs well, comparison with commercial ultrasound systems revealed that the acoustic tracking approach still has the following limitations. The tracking accuracy is determined by measuring the minimum horizontal distance that causes the array to be indistinguishable between two adjacent positions. The sham probe was fixed on the motor to sweep the receiver unit at a step setting from 0.001 to 1 mm. The experimental results show that 0.7 mm is the minimum horizontal distance between two adjacent positions that can be tracked using the acoustic approach. In other words, the simulation system may display the same image even when the sham probe is moved by up to 0.7 mm. The accuracy of the probe angle was also measured, by inclining the probe from 0.2° to 2°. The experimental results show that a resolution of 0.5° is achievable. In a trial involving sonographers with 10 years of experience in ultrasound examinations, they considered that the moving speed and sensitivity of the sham probe kept up with the images display on the simulation system, and hence provided an accurate simulation of a real examination.

Since many studies have used the OT [24] and EMT [18–20] approaches for ultrasound simulation systems, the performance of the presented acoustic tracking approach was compared to those of the two other approaches (Table 1). Most of the OT and EMT systems used in these studies were developed by the same company (Northern Digital Inc., Waterloo, Canada). The Polaris Spectra, an OT system, provides the best tracking accuracy (of 0.25 mm), and the distance from the markers to the camera varies between 95 and 240 cm. The Polaris Vicra also provides a tracking accuracy of 0.25 mm, with distances from the markers to the camera of between 55.7 and 133.6 cm. Two types of EMT system based on an electromagnetic field generator are used in medical ultrasound simulation systems. For a planar field generator, the tracking accuracies were 0.7 and 0.48 mm and the angle accuracies were 0.2° and 0.3° for sensors with five and six DoF, respectively; the corresponding values for a tabletop field generator were 1.2 and 0.8 mm and 0.5° and 0.7°. The accuracy of the presented acoustic tracking approach falls between those of the OT and EMT approaches. However, as mentioned above, the acoustic approach overcomes some important disadvantages of the OT and EMT approaches, such as the line-of-sight problem and electromagnetic interference. Acoustic tracking methods may be influenced by the environment. For example, the sound velocity was influenced by the temperature and humidity in the air. However, we did not use sound velocity as the parameter to determine the probe position. On the contrary, the ultrasonic attenuation (we detect the amplitude of receiving signals) was used in our system. If the operation of this system was performed under a room temperature with a stable humidity, the effect of environment for the acoustic tracking accuracy is limited in this study. Therefore, combination of acoustic tracking approach and other approaches (OT or/and EMT) for developing ultrasound simulation system should be considered in the future works.

**Table 1:** Comparison of the system performances between the acoustic tracking approach, OT, and EMT

OT systems by northern digital inc.	
System	
Polaris Spectra^®^	Tracking accuracy: 0.25 mm from 95 to 240 cm Tracking accuracy: 0.3 mm from 240 to 300 cm
Polaris Vicra^®^	Tracking accuracy: 0.25 mm from 55.7 to 133.6 cm

EMT systems by Northern Digital Inc.	
Planar field generator	
Five-DoF sensor	Tracking accuracy: 0.7 mm Angle accuracy: 0.2°
Six-DoF sensor	Tracking accuracy: 0.48 mm Angle accuracy: 0.3°
Tabletop field generator	
Five-DoF sensor	Tracking accuracy: 1.2 mm Angle accuracy: 0.5°
Six-DoF sensor	Tracking accuracy: 0.8 mm Angle accuracy: 0.7°

Acoustic tracking approach	
Five transmitting and 25 receiving elements	Tracking accuracy: 0.7 mm Angle accuracy: 0.5°

## Conclusion

This study investigated a novel acoustic tracking approach for an ultrasound simulation system. Air-coupled ultrasound elements are key components in this approach. Based on the acoustic signals received from a sham ultrasound probe, the position and angle of the moving sham probe can be detected precisely in this system, and the corresponding ultrasound image is displayed simultaneously on the screen. The system performance was verified using three different subjects, with the results showing that the dynamic images from the simulator perfectly match those from an actual clinical ultrasound system. The tracking and angle accuracies of the presented acoustic tracking approach were 0.7 mm and 0.5°, respectively. Future studies should focus on constructing a database of clinical ultrasound images, particularly for rare disorders.

## Electronic supplementary material

Below is the link to the electronic supplementary material.

Supplementary material 1 (AVI 35343 kb)

Supplementary material 2 (AVI 35226 kb)

Supplementary material 3 (AVI 35226 kb)
